# Injectable Hydroxyapatite and Tetracalcium Phosphate Granules Combined with Autologous Bone Marrow Concentrate: Toward Shape-Adaptive, Single-Step Bone Regeneration

**DOI:** 10.3390/jfb17090456

**Published:** 2026-09-08

**Authors:** Melania Maglio, Maria Sartori, Annapaola Parrilli, Lucia Martini, Enrico Lucarelli, Michela Pierini, Chiara Bellotti, Marzio Piccinini, Susanna Prosperi, Milena Fini, Gianluca Giavaresi

**Affiliations:** 1 Surgical Sciences and Technologies, IRCCS Istituto Ortopedico Rizzoli, Via di Barbiano 1/10, 40136 Bologna, Italy; melania.maglio@ior.it (M.M.); lucia.martini@ior.it (L.M.); gianluca.giavaresi@ior.it (G.G.); 2 Empa—Swiss Federal Laboratories for Materials Science and Technology, 8600 Dübendorf, Switzerland; annapaola.parrilli@empa.ch; 3 Osteoncology, Bone and Soft Tissue Sarcomas and Innovative Therapies Unit, IRCCS Istituto Ortopedico Rizzoli, Via di Barbiano 1/10, 40136 Bologna, Italy; enrico.lucarelli@ior.it (E.L.); michela.pierini@ior.it (M.P.); chiara.bellotti@ior.it (C.B.); 4 Lincotek Medical, Via al Dos de la Roda, 60 Pergine Valsugana, 38057 Trento, Italy; marzio.piccinini@lincotek.com (M.P.); susanna.prosperi@lincotek.com (S.P.); 5 Scientific Direction, IRCCS Istituto Ortopedico Rizzoli, Via di Barbiano 1/10, 40136 Bologna, Italy; milena.fini@ior.it

**Keywords:** hydroxyapatite/tetracalcium phosphate, injectable bone substitute, calcium phosphate ceramics, bone marrow concentrate, alginate coating, bone regeneration, bone graft substitute, histomorphometry, sheep model

## Abstract

Background: Injectable bone graft substitutes offer a practical alternative to preformed ceramic scaffolds by adapting to irregular bone defects while preserving osteoconductive properties. This study investigated the in vivo performance of hydroxyapatite/tetracalcium phosphate (HA/TTCP) granules, uncoated or alginate-coated, with or without autologous ovine bone marrow concentrate (oBMC), in a standardized sheep bone defect model. Methods: oBMC was characterized for cellular composition, viability, and recovery. Implanted HA/TTCP formulations were evaluated after 6 and 12 weeks by micro-computed tomography and histomorphometry. Results: Uncoated HA/TTCP granules promoted bone formation, integrating with host bone. Alginate coating improved injectability and handling but influenced granule organization and degradation. Uncoated HA/TTCP + oBMC showed a more favorable response than the coated formulation, with higher bone-to-implant contact (*p* = 0.004) and bone volume (*p* = 0.031) at 12 weeks. A modest increase in peri-implant bone volume was observed from 6 to 12 weeks in the HA/TTCP + oBMC group, though this did not retain statistical significance once clustering was accounted for. No significant direct effect of oBMC was observed compared with the cell-free formulation. Conclusions: Injectable HA/TTCP granules + oBMC represent a promising single-step strategy for irregular bone defects, although further coating optimization is needed to preserve biological performance while maximizing handling advantages.

## 1. Introduction

Since their introduction in the biomedical field more than fifty years ago, ceramics have established themselves as reference biomaterials in orthopedics, starting from prosthetic surgery, thanks to their characteristics of biocompatibility and resistance to wear. Over the years, the applications of ceramic materials have expanded, especially for calcium phosphate (CaP)-based bioceramics, and many of them, such as β-tricalcium phosphate (β-TCP) and hydroxyapatite (HA), are already widely applied in clinical practice [1,2]. In Europe, it is estimated, for example, that on average 65% of hip arthroplasties use a ceramic head, while approximately 6% also use ceramic liners [3].

The efficiency of ceramic materials in orthopedics is to be found in their composition and in their peculiar chemical-physical characteristics. First, the ability of these materials in promoting cells adhesion and proliferation relies on the perfect mimicking of bone tissue, considering that more than two-thirds of the inorganic phase of bone is made of CaP compounds, thus showing excellent biocompatibility, osteoconductivity and osteoinductivity properties [4,5].

Another peculiar aspect of ceramics contributing to a good outcome for bone regeneration is the release of inorganic ions during the degradation phase of the material. Inorganic ions act as biological stimuli, creating a bioactive environment suitable for stimulating the biological tissues surrounding the implant towards regeneration [6]. HA and TCP materials are known for their ability to release calcium (Ca^2+^) and phosphate (PO_4_^3−^) ions, key players for bone deposition and mineralization, as bone itself is mainly composed of HA, a crystalline form of calcium phosphate. Specifically, calcium ions stimulate various cellular processes, including the differentiation of mesenchymal stem cells into osteoblasts. Furthermore, calcium is involved in the regulation of bone morphogenesis, contributing to the formation of new bone tissue and strengthening the extracellular matrix. Phosphate ions promote the precipitation of HA crystals, improving the formation of bone minerals. The release of phosphate also helps in the process of bone mineralization, that is, the incorporation of minerals (mainly calcium) into the organic matrix, a fundamental step in making the bone stronger and more stable [7].

The process of ionic dissolution via the release of ions from ceramic materials is generally influenced by factors such as the pH of the microenvironment and the porosity of the material, which allows for greater contact with biological fluids and facilitates release, and its chemical composition, which determines the type and number of ions released [8]. In this context of active biological interaction, the incorporation of cellular adjuvants, such as bone marrow–derived cells, can significantly improve the osteogenic response by providing both progenitor elements and signaling factors that stimulate bone tissue formation [9].

Despite these advantageous features, some challenges remain for the comprehensive clinical use of bioceramic materials, especially for the treatment of large defects. The intrinsic brittleness of many bioceramics impairs their mechanical performance, allowing crack propagation, which may compromise implant stability, thus limiting their use in areas subject to dynamic mechanical loads. In addition, the degradation rate should be strictly coupled with bone regeneration to guarantee the preservation of the tissue’s structural integrity while allowing bone integration and growth [10]. A peculiar characteristic of some ceramic materials, which gives them a versatility that can overcome some of the aspects mentioned above, is their injectability, that is, the ability to be extruded through a syringe. Their two-phase composition, consisting of a liquid phase and a powder phase that determines the formation of a paste, enables extrusion. However, this same feature may also lead in some cases to the onset of phenomena called filter pressing, which involves a separation of the two phases upon application of the pressure gradient. This results in a partial extrusion of the paste and, more importantly, in an altered composition of the material that is injected. Injectability is mainly related to the material’s rheological properties and, in general, to specific factors such as viscosity, the granulometry of the ceramic powders, and their cohesion [11].

This latter aspect is particularly challenging because it is related to the ability to preserve geometrical integrity during cement setting, allowing extrudability but preventing, at the same time, uncontrolled CaP particle release, which might have side effects on biocompatibility. Acting on cohesion properties requires the addition to the cement of elements that can increase the viscosity in the overall formulation, like biodegradable polysaccharide gel that, upon covering the cement and cross-linking in the presence of a saline solution, can ameliorate the cohesion of the granules, limiting their dispersion during surgery and after implantation [12].

Among water-soluble polymers, alginate, a polysaccharide derived from the cell wall of brown seaweed or algae, is widely used in various clinical applications, thanks to the lack of immunogenicity and toxicity and the low cost. Alginate has been proven to be biocompatible, allowing osteoblast cell growth and proliferation and, thanks to the ability to form jelly-like substances when in contact with calcium ions, can create a three-dimensional network serving as carriers or scaffolds [13].

The evolution towards new formulations, potentially enriched with cells or growth factors and natural polymers, opens new scenarios for improving clinical use that, although consolidated, is not always able to meet specific needs related to patient comorbidities or complex defects. In particular, the treatment of irregular or anatomically complex bone defects remains challenging, as preformed grafts or scaffolds may require intraoperative shaping and may not completely fit the defect geometry. In this context, injectable and granular biomaterials may offer an important clinical advantage, as they can be delivered directly into the defect and conform to its three-dimensional shape, potentially simplifying intraoperative handling. In addition, combining an osteoconductive ceramic phase with an autologous biological component may therefore provide both a structural substrate for new bone formation and a local biological stimulus supporting tissue regeneration. From a clinical perspective, this approach may be particularly relevant when the objective is to combine material delivery and biological augmentation within a single surgical procedure. However, this shape adaptability must be accompanied by sufficient cohesion and stability of the material after implantation to prevent uncontrolled particle dispersion and preserve the intended distribution of the graft within the defect.

Therefore, the aim of the present study was to evaluate the biocompatibility and osteogenic properties of a new bone substitute in the form of granules made of HA/TTCP alone and HA/TTCP granules coated with alginate in a sheep model of bone defect, also considering the influence of the addition of ovine bone marrow concentrate (oBMC), obtained from bone marrow harvesting. Among calcium phosphate ceramics, tetracalcium phosphate (TTCP) stands out for its high Ca/P ratio (2.0), which makes it the most basic and reactive CaP phase. Its dissolution contributes to creating a favorable ionic microenvironment that favors apatite precipitation and supports early-stage mineralization. In addition, under physiological conditions, TTCP can convert in situ into HA, thus ensuring a gradual transition from the synthetic scaffold to a bone-like mineral phase, providing a biologically active surface that enhances protein adsorption, cell adhesion, and osteoblast activity [14,15].

## 2. Materials and Methods

### 2.1. HA/TTCP Granules and Gel-Coated HA/TTCP Granules Preparation

Briefly, synthetic bone substitutes in paste based on HA/TTCP granules were provided by Eurocoating SpA (now Lincotek Medical, Trento, Italy). The granules were spherical, with a diameter ranging from 300 to 600 μm, and contained HA and TTCP in a 60:40 weight ratio. According to the manufacturer’s specifications, the granules had a surface microporosity of 0.1–10 μm, uncoated and coated with dried alginate. Extensive materials characterization and cytocompatibility have been previously described [16].

### 2.2. In Vivo Study

In vivo studies were performed in compliance with Italian laws on animal experimentation, upon approval by the Ethics Committee of the IRCCS Rizzoli Orthopaedic Institute and Ministry of Health on 24 October 2012 (Prot.0039666 of 19 November 2012) To ensure the quality of the study, the in vivo research methodology has been reported according to criteria set in the Animal in Research: Reporting in Vivo Experiments (ARRIVE) guidelines [17]. Eight crossbred sheep (breeding: Pancaldi Raffaele, Budrio, Bologna, Italy), 50 ± 8 kg body weight, were housed in individual cages and fed a standard diet, hay and water ad libitum. After a quarantine period of 5 days, the animals were submitted to surgery for experimental material placement in the trabecular bone tissue. The animals were pre-medicated with intramuscular injections of 10 mg/kg ketamine and 0.2 mg/kg xylazine, and a subcutaneous injection of 0.0125 mg/Kg atropine sulfate. Then, an intravenous injection of 10 mg/Kg thiopental sodium was performed to induce general anesthesia. During surgery, anesthesia was maintained with an O_2_/air (60/40 L/min) mixture and 2–3% sevorane with assisted ventilation. Upon awakening, 62.5 mL/mg of methylprednisolone sodium succinate was administered intravenously.

Postoperatively, antibiotic and analgesic therapies were administered, and the animals were not restricted in their movements. To monitor the dynamics of bone ingrowth, the animals received i.m. injections of 30 mg/kg b.w. oxytetracycline fluorochrome 2 days on, 10 days off and 2 days on, 18 days before the end of the experimental period.

At the end of each experimental period (6 and 12 weeks), 4 animals underwent euthanasia under general deep anaesthesia as previously described, with the intravenous administration of 20 mL of a specific veterinary euthanasic drug.

### 2.3. Surgical Procedures

#### 2.3.1. Bone Marrow Harvesting and Bone Marrow Concentrate Preparation

After general anaesthesia, the animals were positioned in prone decubitus, and bone marrow was harvested from both iliac crests using a needle and a heparinised syringe. Samples were taken at multiple sites to obtain a total volume of approximately 60 mL. Then, BMC, consisting of the mononuclear cell fraction containing the mesenchymal stromal cells (MSCs), was obtained with an RSQ-60 system (Thermogenesis), using the IOR-G1 device supplied by Novagenit Srl (Mezzolombardo, Italy), obtaining a volume of approximately 6 mL.

#### 2.3.2. Graft Preparation

The HA/TTCP and alginate gel-coated HA/TTCP granules were supplied in 1 mL sterilized prefilled syringes, each containing 600 mg of sample (0.5 cc). During the bone defect surgery, the materials were prepared. In detail, for the preparation of polysaccharide gel-covered HA/TTCP granules combined with oBMC, 200 µL of oBMC was diluted with cross-linking solution (1:1) in a sterile vial. The cross-linking solution is composed of 0.06 M calcium chloride (CaCl2, Carlo Erba, Milano, Italy) in demineralized water. After blending the composite, the contents (400 µL) were picked up and inserted into a pre-loaded gel-covered granules syringe, paying attention to completely wet all granules. For the preparation of the material used as a reference, 400 µL of cross-linking solution was added to a pre-loaded gel-covered HA/TTCP granules syringe, and the same procedure was followed. For the preparation of the HA/TTCP granules, 300 µL of oBMC was added to a pre-loaded HA/TTCP granules syringe. The sample control was prepared by adding 300 µL of saline solution to a pre-loaded HA/TTCP granules syringe.

#### 2.3.3. Graft Implantation

The animal was then placed alternatively on each side, thus enabling access to the medial surface of the proximal epiphysis of the left tibia and to the lateral surface of the right femoral condyle, and then to the proximal epiphysis of the right tibia and the lateral surface of the left femoral condyle. Through drill bits with a progressive increase in diameter, from 2 to 8 mm (electric motor for orthopedic surgery PEN DRIVE, DePuy Synthes Srl, Johnson & Johnson Medical, Roma, Italy), bone defects of 8 mm in diameter and a depth of about 10 mm were created in each of the sites. The materials HA/TTCP and gel-coated HA/TTCP, with or without oBMC, were extruded and inserted into the bone defect 5 min later for the cross-linking of the polysaccharide gel. The experimental material, gel-coated HA/TTCP, was mixed with 0.4 mL CaCl2—used as a setting accelerator—or with the association of 0.2 mL of oBMC and 0.2 mL of CaCl2 (gel-HA/TTCP+oBMC) before implantation. The experimental design and allocation of formulations to implantation sites are shown in Table S1 (Supplementary Materials).

### 2.4. In Vitro Cell Evaluation

Cell count and viability of mononuclear cells (MNCs) were assessed for each sample of ovine native bone marrow (oBM) and concentrated bone marrow (oBMC) using the NucleoCounter^®^ NC-100™ (ChemoMetec A/S, Allerød, Denmark) following the manufacturer’s protocol based on propidium iodide (PI) uptake and fluorescence microscopy. The efficiency of the MNC enrichment technique was evaluated and expressed as a percentage.

MSCs were isolated from oBM and oBMC samples through Ficoll gradient centrifugation as previously described [18]. Cells were seeded at 2 × 10^5^ nucleated cells/cm^2^ in 150 cm^2^ culture flasks (Euroclone) and maintained in α-modified minimum essential medium (α-MEM; BioWhittaker, Lonza, Euroclone, Milano, Italy) supplemented with 10% lot-selected fetal bovine serum (FBS; Lonza), 1% GlutaMAX™ (Invitrogen, Thermo Fisher Scientific, Milano, Italy), and 1% penicillin-streptomycin (Sigma-Aldrich, Merck, Milano, Italy) in standard culture conditions. MSCs were identified by their adherent, spindle-shaped morphology and proliferative capacity. At 70–80% confluence, cells were detached using mild trypsinization (TripLe™ Select; Life Technologies, Thermo Fisher Scientific, Milano, Italy), counted via NucleoCounter, and reseeded at 2000 MSC/cm^2^ for up to 12 passages. Cell kinetics, including doubling time (DT) and population doublings (PDs), were calculated at each passage. The cumulative population doubling (CPD) was determined as the sum of PDs over passages.

The Colony Forming Unit (CFU) assay was performed to assess progenitor cell yield and estimate MSC frequency in oBM and oBMC samples. Duplicate aliquots of MNCs were plated at low density (1.5 × 10^5^ cells) in 10 cm culture dishes (Falcon, Becton Dickinson), and cultures were maintained for 14 days under the above-described conditions. Cells were then fixed with methanol for 10 min at room temperature and stained with 1% methylene blue in 0.01 M borate buffer (pH 8.5). MSC colonies, each derived from a single progenitor, were enumerated (*n* = 7).

### 2.5. Microtomographic Analyses

After euthanasia, explanted bone segments containing the different material combinations were immediately fixed in buffered 4% paraformaldehyde for 48 h and then dehydrated in ethanol solution at increasing percentages up to 100%, for 48 h per step. Prior to the resin inclusion, samples randomly selected for micro-CT analysis were scanned with the high-resolution microtomography system Skyscan 1172 (Bruker Micro-CT, Kontich, Belgium) at a source voltage of 100 kV and a source current of 100 μA, with a 0.5 mm aluminum filter and a nominal voxel size of 14 μm. Each sample was rotated through 180° with a rotation step of 0.4° and a frame averaging of 2. The images obtained from the acquisition were reconstructed by the software NRecon (version 1.6.8.0) with corrections for alignment, depending on the acquisition, beam hardening and ring artifact reduction. The resulting images were exported as jpg files of 2000 × 2000 pixels, with a pixel size of 14 μm.

**Figure 1:**
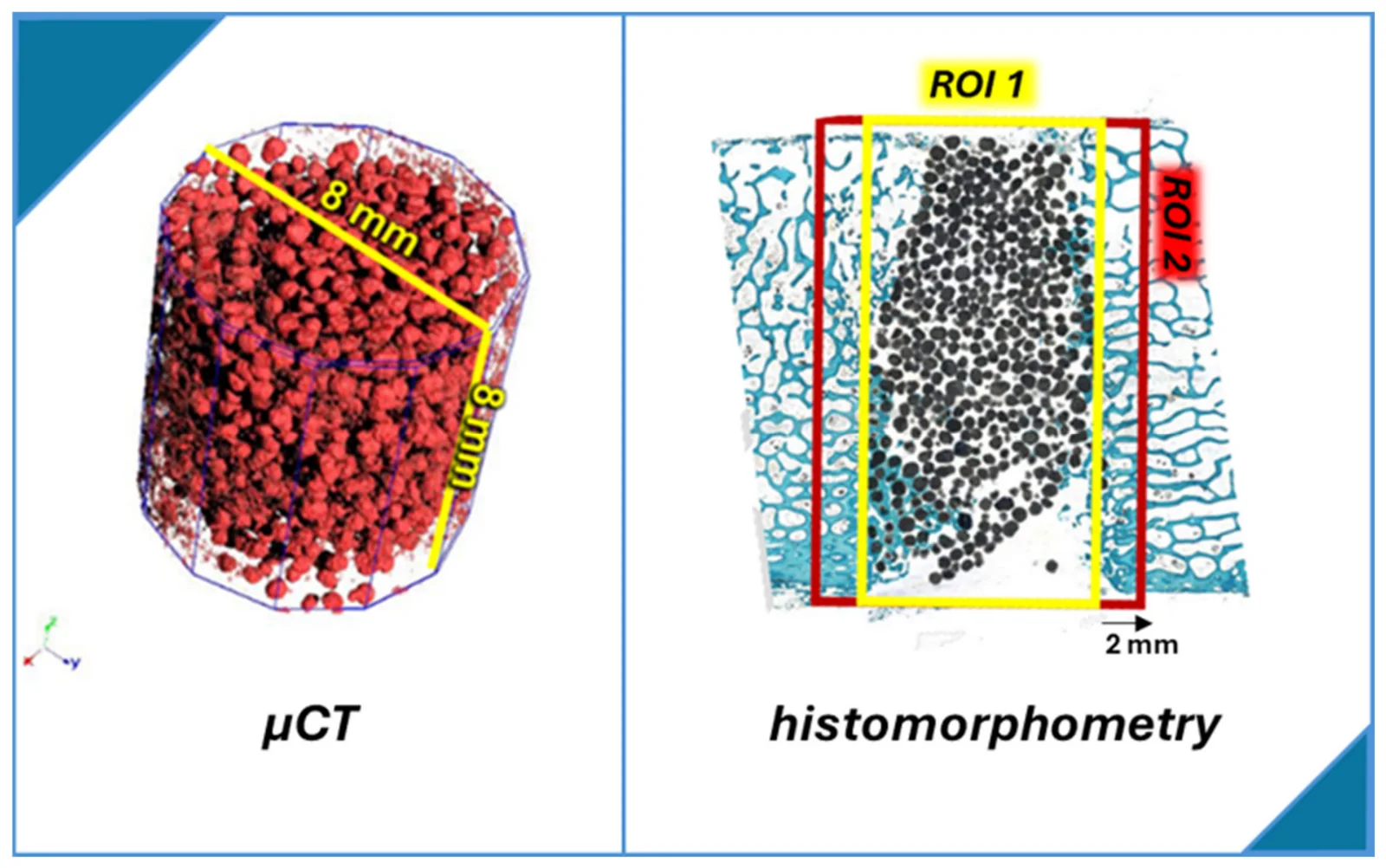
Representative images of the VOI for the morphometric micro-CT analysis of the HA/TTCP materials (in red) and of the ROI for the histomorphometric measures on bone and HA/TTCP materials: in yellow, ROI 1, corresponding to the bone defect; in red, ROI 2, corresponding to the peri-implant bone around the defect area; trabecular bone tissue at the implant and peri-implant sites appeared blue-stained.

Quantitative 3D analyses were carried out with CTAn software (version 1.13, Bruker Micro-CT, Kontich, Belgium). A cylindrical volume of interest (VOI) with a diameter of 8 mm and height of 8 mm was defined to evaluate the material in the surgically created defect (Figure 1).

The following morphological parameters were evaluated:

- 2D Material Granule Diameter Mat.p.ECD (in mm), expressed as the surface-equivalent circle diameter;
- 3D Material Granule Diameter (in mm), expressed as the volume-equivalent sphere diameter (Mat.p.ESDA).

### 2.6. Histology and Histomorphometrical Evaluation

After dehydration, the samples were embedded in resin based on methyl methacrylate (Merck, Schuchardt, Milano, Italy); once polymerized, samples were cut along the major axis of each implant with the Exact cutting system (EXAKT Cutting Systems, Norderstedt, Germany) and the obtained sections were thinned up to 20 ± 10 µm, polished and stained with Toluidine Blue, Acid Fuchsin and Fast Green. Images of the stained sections were acquired by a high-resolution scanner for digital pathology (Aperio ScanScope CS, Leica Biosytem, Nussloch GmbH, Nussloch, Germany) and then used for the evaluation of bone tissue and osteointegration through QWIN software imaging analysis (version 3.5.1, Leica Microsystems, Nussloch, Germany).

Two rectangular regions of interest (ROI) were identified and measured for each implant site (Figure 1):

- ROI 1, corresponding to the bone defect, for the evaluation of the material and of the newly formed bone inside the defect.
- ROI 2, corresponding to the area around the defect extended to about 2 mm in the radial direction, for the evaluation of the peri-implant bone.

The following histomorphometric parameters evaluated in ROIs were measured in accordance with the *Histomorphometry Nomenclature by the Committee of the American Society for Bone and Mineral Research* [19]:

- Bone to implant contact (BIC%): Percentage of the implant perimeter within the defect that was in direct contact with newly formed bone, calculated as the ratio between the bone–implant contact perimeter and the total implant perimeter within the analyzed ROI × 100;
- Bone volume fraction (BV/TV%): Proportion of the analyzed tissue volume occupied by trabecular bone, calculated from the bone area (B.Ar) relative to the total tissue area (T.Ar) of the selected ROI—Material volume fraction (Mat.V/TV %), proportion of the analyzed tissue volume occupied by residual material, calculated from the material area (Mat.Ar) relative to the total tissue area (T.Ar) of the selected ROI Unstained sections for each implant were used for the measures of dynamic histomorphometry through semi-automatic optical microscope Olympus BX51 connected to the Olympus XC50 digital camera and the CellSense system of image analysis (Olympus Optical Co. Europe GmbH, Hamburg, Germany). The parameters evaluated were as follows:
- Mineral apposition rate (MAR, uM/day): The rate of new mineral deposition, calculated from the distance between the fluorescent labels divided by the time interval between the corresponding labeling events—Mineralizing Surface (MS/BS, %): The proportion of bone surface undergoing mineralization, calculated as the double-labeled surface plus one-half of the single-labeled surface relative to the total bone surface;

All histomorphometric, micro-CT, and CFU analyses were performed under blind conditions with respect to treatment allocation. Samples were identified only by animal number and anatomical implantation site, without disclosure of the corresponding material formulation. Treatment allocation was decoded only after completion of the outcome assessments.

### 2.7. Statistical Analysis

Statistical analysis was performed using SPSS v.12.1 (SPSS Inc., Chicago, IL, USA). Data were reported as mean ± standard deviation (SD) at a significance level of *p* < 0.05. The nonparametric Kruskal–Wallis (KW) test was performed for comparisons between independent data, followed by the Mann–Whitney test (U) for multiple comparisons using the Bonferroni correction of the significance value (value of α/number of comparisons between groups: 0.05/6; *p* = 0.0083). A post hoc sensitivity analysis addressing within-animal clustering, prompted by a deviation from the planned allocation (see Section 3.1), is described in Section 3.4.

## 3. Results

### 3.1. Surgical Procedures

All surgeries were carried out without complications. Due to limitations encountered during bone marrow collection, the planned balanced allocation could not be maintained in the 6-week cohort. While one case received each of the four formulations once, in the other three, two implantation sites received the same formulation, resulting in an unbalanced allocation across the 6-week cohort. The allocation at 12 weeks remained fully balanced. Differences in plasticity and implant mechanics were observed in the experimental materials HA/TTCP and gel-covered HA/TTCP, with and without oBMC. Gel-covered HA/TTCP was found to be more homogeneous and easier to extrude, whereas reference material HA/TTCP granules required higher extrusion pressure at the time of implantation due to the material’s tendency to compact inside the syringe. In addition, a greater dispersion from the defect area towards the outer surface of the treated bone segment was observed for the granules of the test material HA/TTCP.

Animals recovered well from surgery and showed no signs of an inflammatory reaction, infection, or scar tissue formation in the implanted sites or lameness.

### 3.2. MSC Cultures and CFU Assays

The nucleated cells contained in oBM and oBMC were expanded in vitro to compare the kinetic parameters. The MSC proliferative kinetics were analysed for four of the sheep that underwent surgery, highlighting no differences in proliferation kinetics between the expanded MSC cultures obtained from oBM and oBMC samples.

**Figure 2:**
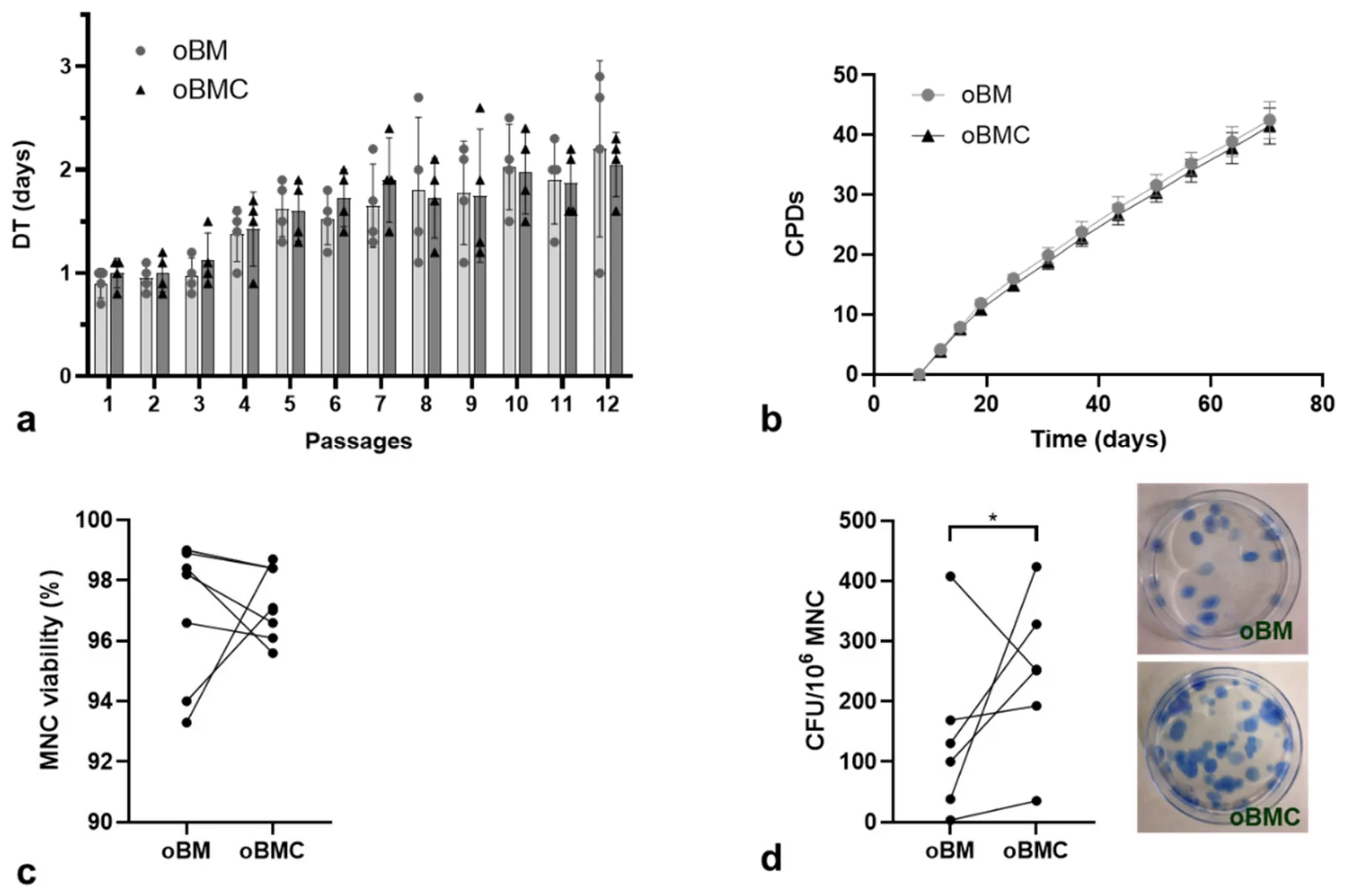
Growth kinetics and clonogenic potential of ovine bone marrow-derived mesenchymal cells (oBM) and ovine bone marrow concentrate-derived mesenchymal cells (oBMC). (**a**) Doubling time (DT) across passages (P1–P12). (**b**) Cumulative population doublings (CPDs) as a function of culture time (days). (**c**) MNC viability in oBM and oBMC preparations. Each point represents an individual animal donor, with paired samples connected by lines. (**d**) Number of MSCs in oBMC, expressed as colonies per million MNC. Individual animal donors are shown as points, with paired samples connected by lines and representative CFU assay images. (*: *p* < 0.05).

Cells obtained from oBMC had the same DT and CPD values as cells obtained from native oBM, suggesting that the system used to concentrate the oBM did not influence the content and the quality of mesenchymal stromal cells, which were maintained. oBM and oBMC samples were then characterized for the content of mesenchymal progenitor cells (MSCs) by performing a CFU assay. The number of MSCs in oBMC, expressed as the number of colonies per million MNCs, was found to be significantly higher than in oBM (*p* < 0.05), with the number of MSCs increased by a factor of 3.8 in oBMC compared to oBM (Figure 2).

### 3.3. µCT Analysis

**Figure 3:**
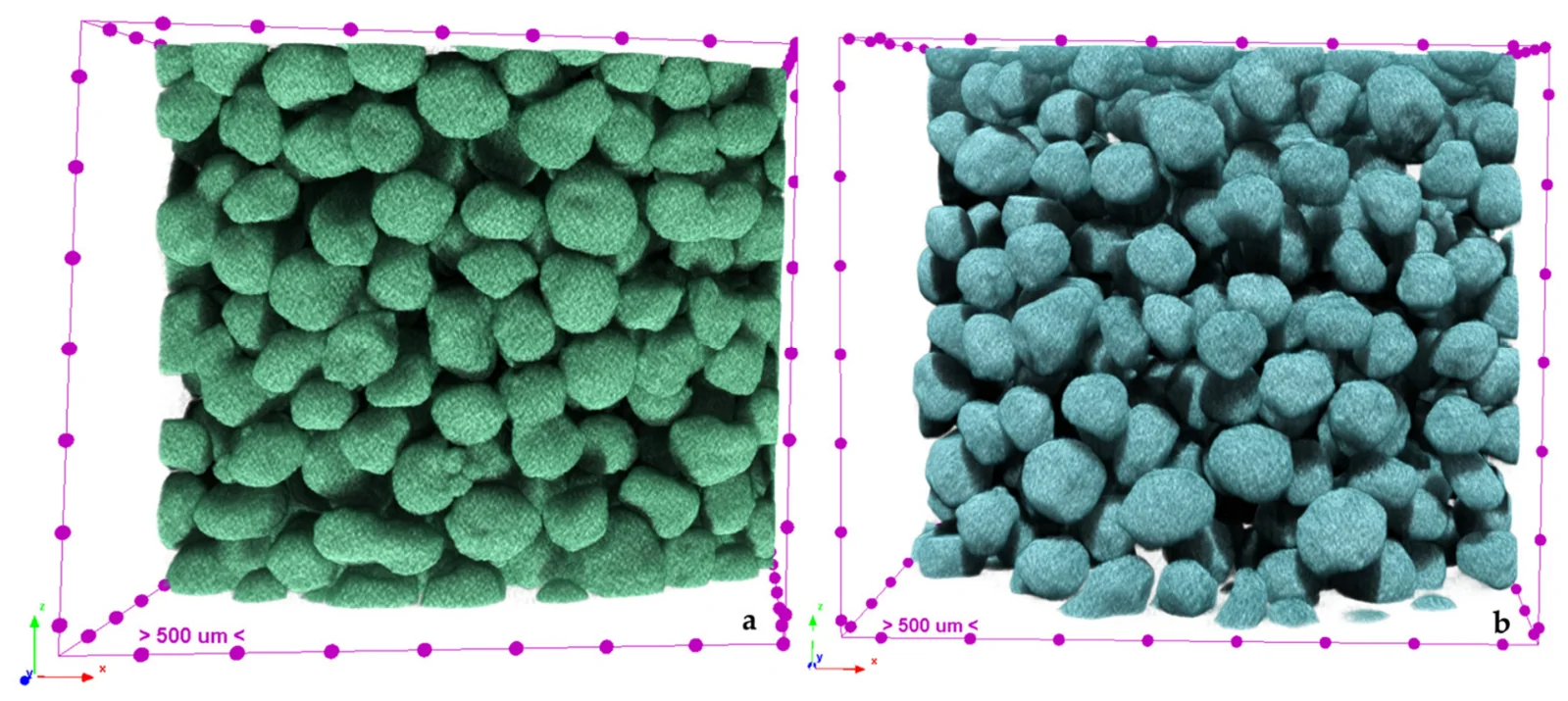
Representative 3D reconstructions of HA/TTCP (**a**) and gel-covered HA/TTCP (**b**), illustrating differences in granule packing and spatial distribution.

After 6 and 12 weeks from implantation, HA/TTCP and gel-covered HA/TTCP are still present, distributed at the implantation site. Gel-covered HA/TTCP granules appeared more dispersed with respect to the HA/TTCP granules (Figure 3).

In all the analyzed samples, the different structures of the trabecular tissue of the two anatomical districts considered (thinner trabeculae in the femoral condyle and thicker and sparser in the tibial plateau) and the apparent difference in granule density (higher in the tibial plateau) are evident, especially in the HA/TTCP material.

**Figure 4:**
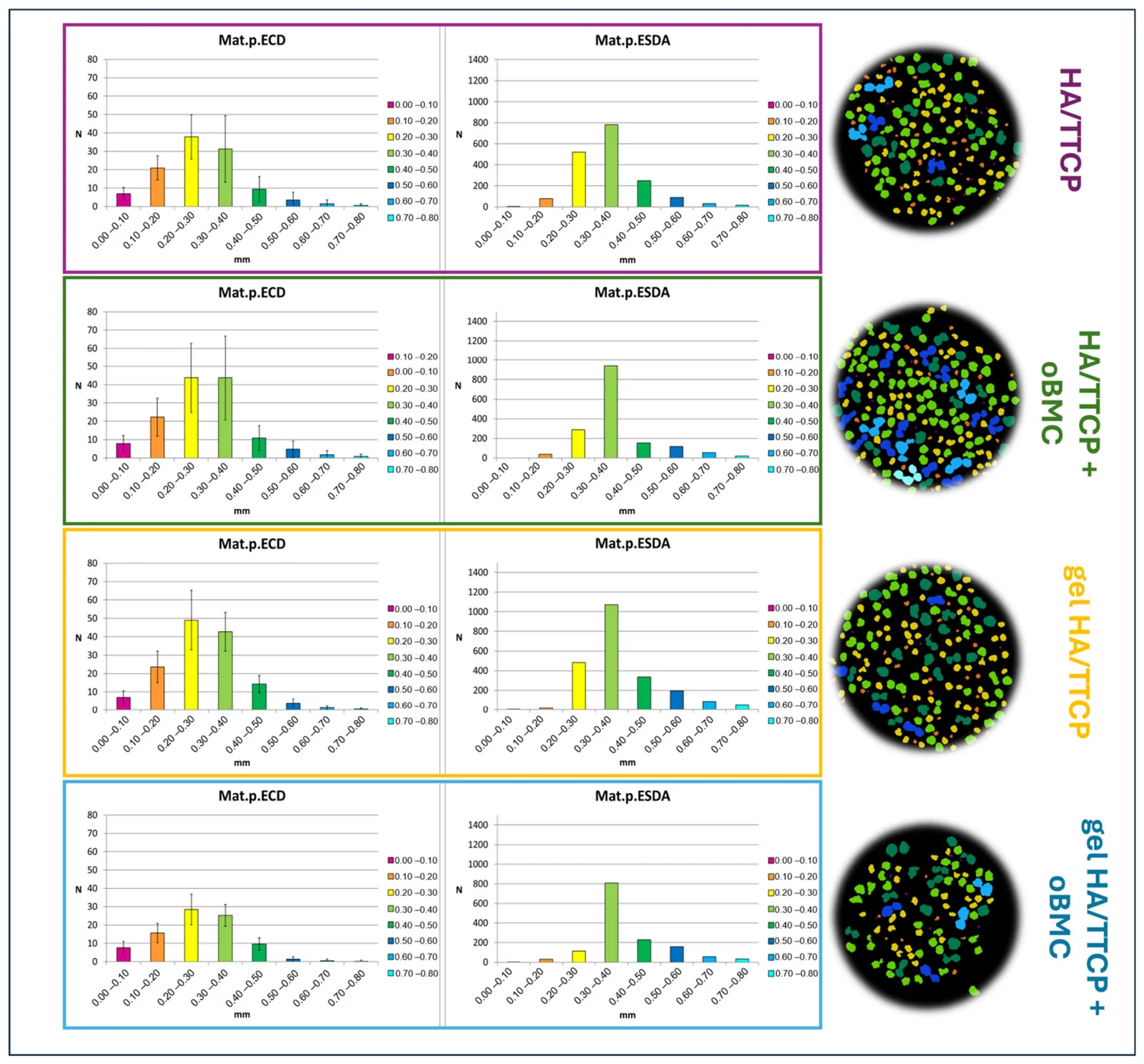
Particle size distribution and 2D reconstruction of HA/TTCP and gel HA/TTCP scaffolds, with or without oBMC. Representative histograms show equivalent circular diameter (Mat.p.ECD) and equivalent spherical diameter (Mat.p.ESDA) distributions.

Morphometric analysis via micro-CT highlighted differences between the groups. The HA/TTCP material exhibited a more uniform Gaussian distribution of the granule diameter intervals than the other materials. In terms of particle size, all formulations showed a prevalence of fragments between 0.20 and 0.40 mm, with a low presence of larger granules in the gel-coated HA/TTCP + oBMC group. Similarly, shape analysis showed a marked peak at approximately 0.30–0.40 mm; the gel-coated HA/TTCP + oBMC formulation showed a pattern consistent with greater fragmentation, characterized by smaller and more spherical particles (Figure 4). These morphological differences could reflect variations in degradation mechanisms and interaction with the host tissue.

### 3.4. Histologic and Histomorphometric Analyses

**Figure 5:**
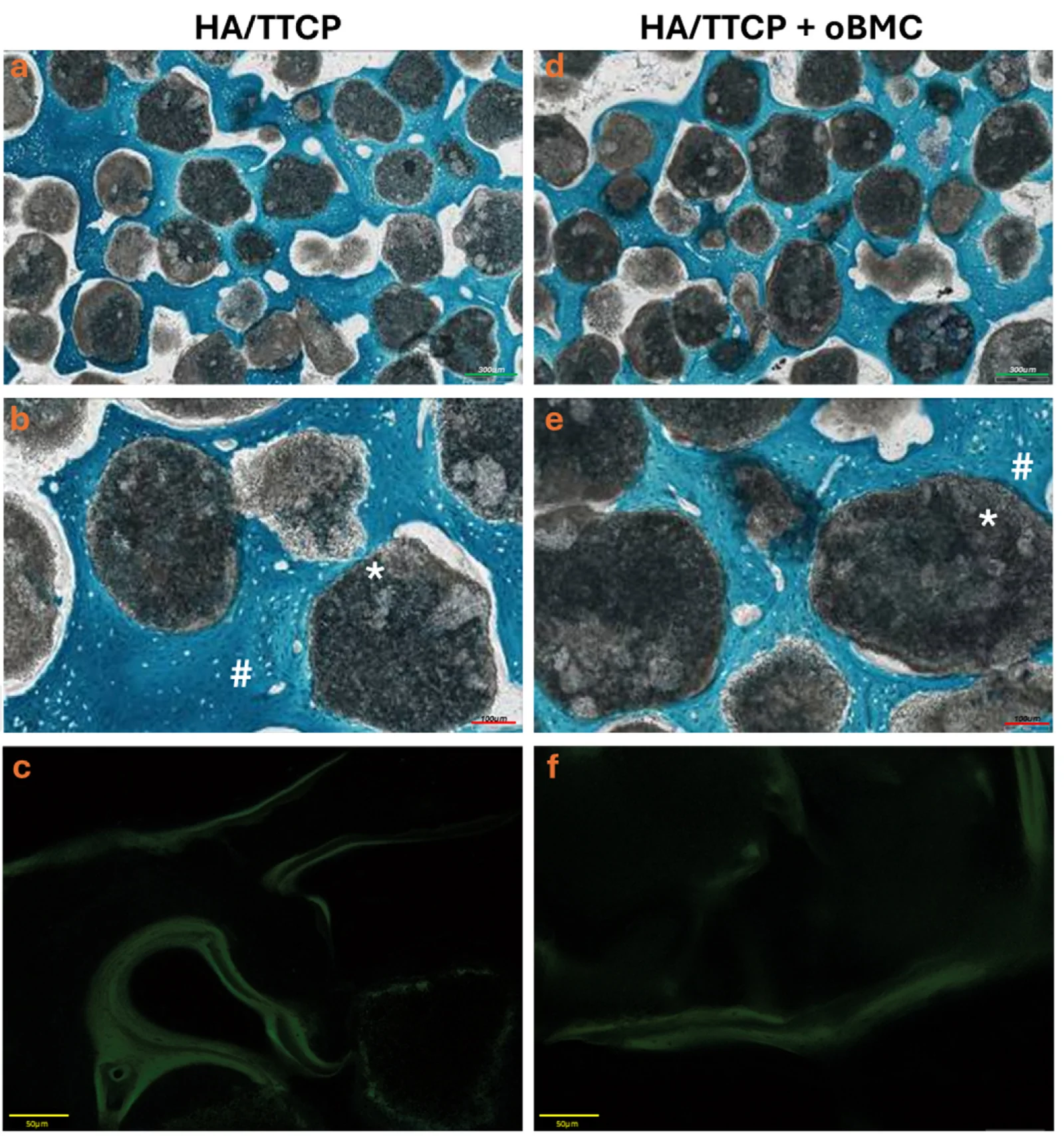
Representative histological and fluorochrome images of HA/TTCP (**a**–**c**) and HA/TTCP + oBMC (**d**–**f**) implants at 6 weeks. Trichrome staining is shown at 8× (**a**,**d**) and 20× (**b**,**e**) magnification; tetracycline labeling is shown at 20× (**c**,**f**). Scale bar 300 µm (**a**,**d**), 100 µm (**b**,**e**), 50 µm (**c**,**f**). Material and newly formed bone are indicated by * and #, respectively. The white areas correspond to intertrabecular marrow spaces surrounding the implanted material.

At both experimental time points, histological evaluations revealed overall positive outcomes for all tested materials and formulations. No signs of adverse reactions were observed. Only in some samples did the lower third of the defect appear incompletely filled by the granules. Bone defects treated with HA/TTCP material showed evidence of new bone formation, both within the material and at the interface with native bone. The granules appeared homogeneously distributed, maintaining a high packing density that favored intergranular contact. Newly formed bone tissue surrounded and incorporated the granules, establishing continuity with the peri-implant bone. In several cases, bone ingrowth and material colonization were so effective that the margins of the defect became histologically indistinguishable. The newly formed tissue closely resembled native bone in both morphology and structure. At the cortical surface, early signs of bone remodeling were observed, along with some occasional leakage of granules outside the defect boundaries (Figure 5).

**Figure 6:**
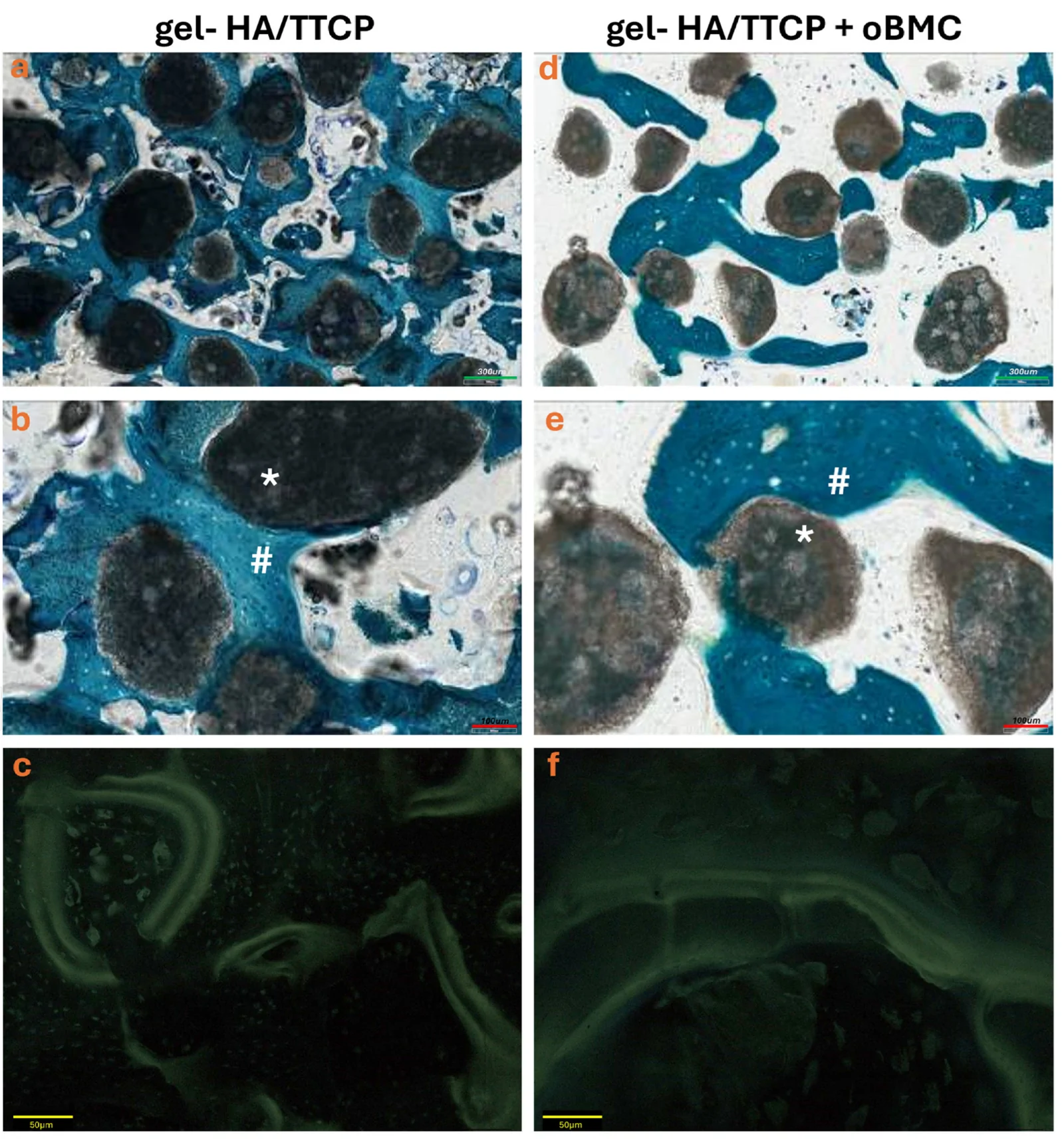
Representative histological and fluorochrome images of HA/TTCP (**a**–**c**) and HA/TTCP + oBMC (**d**–**f**) implants at 12 weeks. Trichrome staining is shown at 8× (**a**,**d**) and 20× (**b**,**e**) magnification; tetracycline labeling is shown at 20× (**c**,**f**). Scale bar 300 µm (**a**,**d**), 100 µm (**b**,**e**), 50 µm (**c**,**f**). Material and newly formed bone are indicated by * and #, respectively. The white areas represent intergranular and intertrabecular spaces, which may include cellular or hematopoietic marrow content and some processing-related empty spaces.

In the gel-covered HA/TTCP group, the alginate coating appeared to influence the spatial arrangement of the granules, which appeared slimmer and more spaced, with reduced density. Bone trabeculae formed primarily in the central area between granules and along the defect periphery. Nonetheless, cortical bone healing was still evident in these samples (Figure 6).

Because three of the four animals in the 6-week cohort received the same treatment at two implantation sites rather than the four distinct formulations originally planned (Section 3.1, Table S1 Supplementary Materials), a post hoc sensitivity analysis was performed to assess the impact of this within-animal clustering on the comparisons described below: site-level values were further averaged within animal for each treatment × time-point combination, yielding one independent observation per animal (*n* = 3–4 per cell), and key pairwise comparisons were repeated on these animal-level data using the Mann–Whitney test. This sensitivity analysis was applied comprehensively to all pairwise comparisons originally found significant in the site-level analysis (20 comparisons across the six histomorphometric variables assessed), not to a subset selected post hoc.

The addition of autologous oBMC is revealed to have a boosting effect on HA/TTCP formulation. At 12 weeks, HA/TTCP + oBMC showed a significantly higher BIC% than gel-coated HA/TTCP + oBMC (*p* = 0.029), indicating enhanced osteoinductive and conductive properties. A modest increase in new bone volume was observed in the HA/TTCP + oBMC group between 6 and 12 weeks (*p* = 0.057). At 12 weeks, this group also outperformed the corresponding gel formulation (*p* = 0.029). The positive trend continued for peri-implant bone, with HA/TTCP + oBMC showing an increasing trend from 6 to 12 weeks (*p* = 0.057), and higher values overall compared to gel groups.

**Figure 7:**
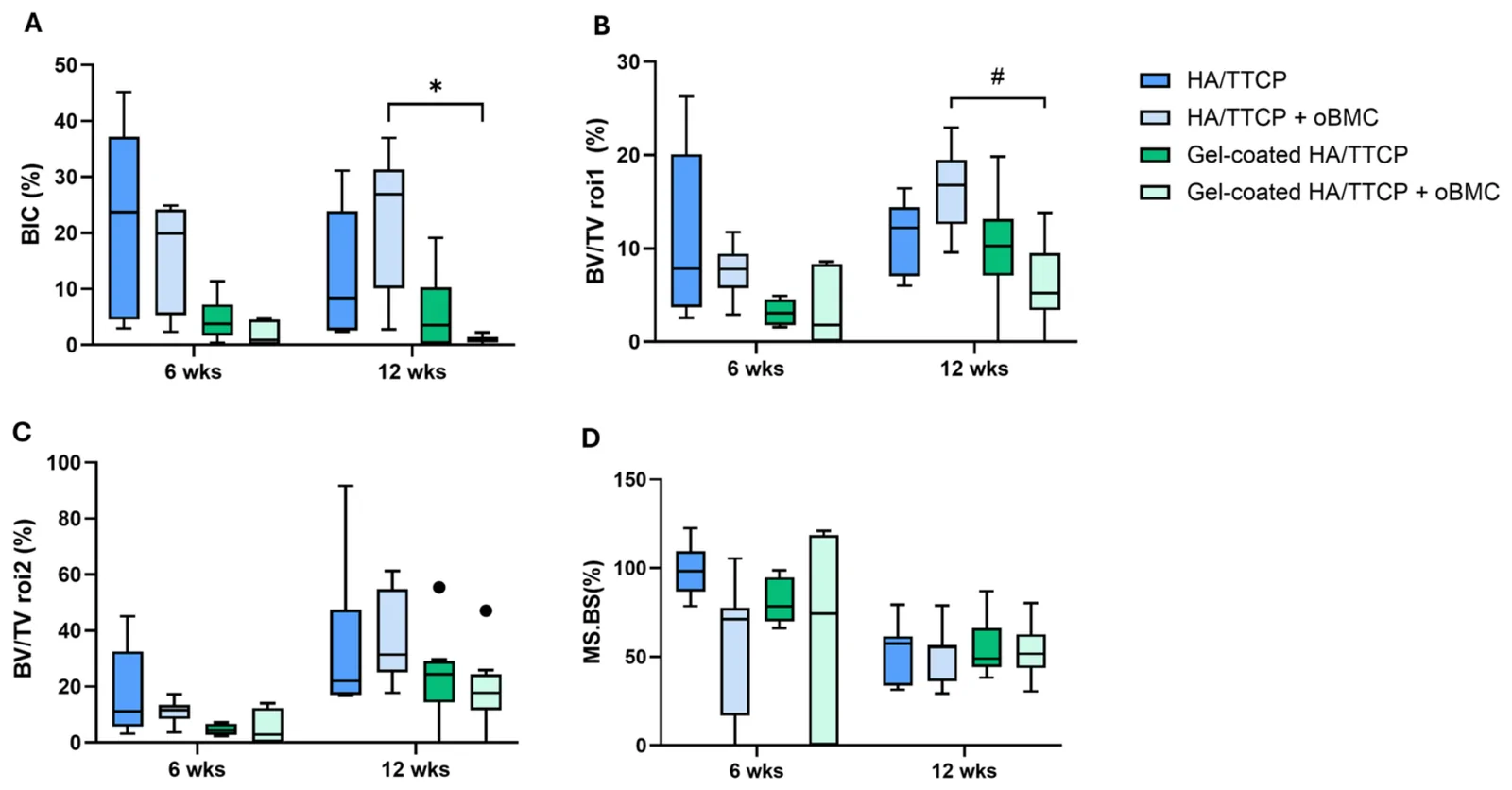
Histomorphometric results of measured parameters at 6 and 12 weeks shown as histograms relative to (**A**) BIC, (**B**) BV/TV of roi1 corresponding to the bone defect, (**C**) BV/TV of roi2 corresponding to the perimplant bone around the defect area, (**D**) MS/BS (• outliers). (**A**) HA/TTCP + oBMC vs. gel-coated HA/TTCP + oBMC 12 (*, *p* = 0.029) at 12 wks; (**B**) HA/TTCP + oBMC vs. gel-coated HA/TTCP + oBMC at 12 wks (#, *p* = 0.029).

Dynamic histomorphometry showed the presence of double fluorescent labels in all samples, indicating active bone apposition and mineralization. However, no statistically significant differences were found between groups for any of the analyzed parameters, except for MS/BS, whose values were slightly higher in HA/TTCP at 6 weeks compared to 12 weeks, though this difference did not reach statistical significance in the animal-level sensitivity analysis (*p* = 0.057) suggesting a modest, non-conclusive trend towards more active bone formation in earlier phases (Figure 7).

## 4. Discussion

The growing trend toward patient-specific solutions has also reached the field of biomaterials—particularly in orthopedic prosthetic applications—exploiting the advances in 3D printing technologies and the increasingly feasible design customization with various biomaterials. However, ceramic materials remain less adaptable to additive manufacturing compared to metals and polymers, due to their intrinsic brittleness, the requirement for high-temperature sintering, and the associated volumetric shrinkage [20]. These aspects lead to higher processing costs due to the need for tailored formulations, specialized equipment, and extensive post-processing, as well as careful optimization to avoid defects and ensure adequate control of porosity and microstructure [21,22]. Interestingly, the extrudability and injectability characteristics of some ceramic formulations, together with the high variety of shapes, do not preclude their use for tailored approaches, thus allowing them to exploit the advantages of their intrinsic bioactive properties by adapting their shape to the implantation site. Unlike structural custom implants, which primarily rely on morphological customization, bioceramics actively support bone regeneration while offering cost-effective, ready-to-use formats adaptable to various clinical needs without complex preoperative planning. Ceramic materials (e.g., injectables or granules) do not require 3D planning or dedicated equipment and can be shaped intraoperatively to conform to the defect, thus resulting in their usefulness in emergency situations, open surgery, or in countries with limited resources. In this sense, ceramics remain a primary field of interest for the biomedical industries, which consistently invest in the development of patentable, scalable and biocompatible formulations [23].

The growing potential of bioceramics for orthopedic applications is reflected in ongoing research efforts, particularly in the preclinical field, focused on developing new formulations and combinations of ceramic materials. This interest in bioceramic formulations shows no signs of waning, highlighting that, despite important milestones already achieved in their orthopedic use, there is still significant room for optimization and a strong drive toward more versatile solutions. The present study has exploited this peculiar characteristic of bioceramic materials, filling a bone defect using a granular formulation that allowed the ingrowth of newly formed bone tissue from the peri-implant site towards the center of the defect. The granular formulation proved well suited to filling the defects created, with a good distribution of the material, which thus created a sort of diffuse spot progressively surrounded by the newly formed bone tissue, in structural continuity with the peri-implant bone. The temporal regeneration pattern suggests the onset of an early regenerative process, which becomes stable over time, as shown by the decrease in MS/BS values from 6 to 12 weeks, and by the significantly higher amount of new bone observed at 12 weeks. The materials appear to support physiological bone regeneration, compatible with in vivo remodeling processes. To maximize the material’s extrudability, an alginate coating was tested. The choice of alginate was based on its ability to increase viscosity and create a cohesive matrix. This allows for uniform coatings or homogeneous pastes, facilitating the production of scaffolds or implants with complex geometries [24]. As a natural polysaccharide derived from algae, alginate is highly biocompatible and well tolerated by the body, supporting the delivery of biological boosting with oBMSCs thanks to the ability to form hydrated gels [25]. As expected, gel-coated HA/TTCP formulations were more easily extrudable and exhibited more homogeneous behavior during surgical manipulation and injection. Despite this promising technical performance, histology showed an impact on the relative density of the grain sizes, with increased spacing between the grains. The presence of alginate in the formulation may have contributed to accelerating the degradation of the ceramic material, presumably by increasing porosity and modifying the local microenvironment, due to ion exchange, swelling, and leaching of the polymer phase. This phenomenon is consistent with what has been reported in the literature for composite systems based on alginate and calcium phosphate [26,27]. This may have modified the geometry and microarchitecture of the paste, also impacting cell adhesion and neovascularization. The too rapid degradation of the matrix could have impaired the structural stability necessary for osteogenic activity, potentially delaying cell adhesion, proliferation and differentiation. Furthermore, as an anionic polysaccharide, alginate may mask the bioactive surface of the ceramic granules, reducing the direct availability of functional groups such as -OH, -PO_4_, and Ca^2+^, which are essential for the material’s bioactivity. This effect may have contributed to the more favorable response observed in the uncoated formulation compared with the coated one, both in the presence of oBMC, suggesting a possible interplay between the ceramic’s surface bioactivity and the biological stimulation provided by the cells, which may be more effective when the ceramic surface is not masked by the coating. The use of BMSCs is well established in bone tissue regeneration approaches thanks to the intrinsic osteogenic capability of these cells, which can differentiate towards the osteogenic lineage, while performing paracrine activity by secreting factors that can promote angiogenesis, proliferation and recruitment of endogenous cells [28].

The more favorable response observed in the uncoated formulation may have been attenuated by the coating in the presence of oBMC, which may have created a physical barrier or altered the microdistribution of the cells. The alginate coating does not entirely inhibit the initiation of new bone formation, as evidenced by the BV/TV results in the regions of interest at both the defect and peri-implant sites, which show a progressive increase over time. Nevertheless, its role as a “temporary barrier”, particularly considering its degradation rate, may not align with the pace of the regenerative process, potentially offsetting some of the surgical advantages.

This evidence is particularly interesting from the perspective of identifying and overcoming potential limits related to the trade-off between surgical handling and biological performance [29,30]. Although the micro-CT morphometric analysis did not reveal statistically significant differences between formulations, the observed tendency toward smaller and more spherical particles in the gel-coated HA/TTCP + oBMC formulation is noteworthy when considered alongside the histological and morphometric findings. This morphological variation may reflect formulation-dependent differences in granule organization and fragmentation and could contribute to the distinct spatial distribution of the material observed histologically. However, given the variability of the measurements, this observation should be regarded as descriptive, and its biological relevance cannot be established from the present data.

To fully exploit the potential of alginate in improving the extrudability of ceramics and thereby facilitating surgical handling, a possible future development could consist of functionalizing it with bioactive peptides (e.g., RGD), capable of promoting cell adhesion and activating osteogenic differentiation pathways. Furthermore, integrating osteoinductive nanoparticles into the coating could help enhance the osteogenic response without altering the system’s manipulability [31].

Other strategies like the inclusion of growth factors (e.g., BMP-2 or VEGF) in a controlled-release manner, or the use of chemical modifications of alginate (e.g., partial oxidation or chitosan grafting) could allow for achieving more efficient cell colonization [32,33]. These approaches could maintain the mechanical and surgical advantages observed in the present study by overcoming the biological limitations found in the in vivo model, without compromising the rationale of the design.

The study also presents opportunities for implementation and improvement to consolidate the results and optimize the experimental setup. First, increasing the sample size would improve the statistical power of the study and provide a more robust assessment of treatment-related differences. A larger number of animals would also help mitigate the impact of practical limitations encountered during bone marrow collection, which resulted in some adaptations to the planned treatment allocation and consequently in an unbalanced distribution of the materials across implantation sites. A sensitivity analysis accounting for this within-animal clustering confirmed the robustness of the principal 12-week finding, while the reported temporal increases did not retain significance once clustering was accounted for, likely reflecting the limited number of independent animals rather than the absence of a true effect consistent with the exploratory nature of this pilot study. A follow-up longer than 12 weeks could provide a more accurate picture of both the degradation rate of the materials and the bone regeneration trend. The integration of histomorphometric measurements with microstructural analysis of the bone tissue through micro-CT imaging would offer a 3D overview of bone growth, also in relation to the distribution of the granules. Implementation with immunohistochemical quantification could help interpret the molecular mechanisms underlying the observed results.

Finally, the design of new formulations could be hypothesized to mitigate some of the effects observed in the coated compositions, through strategies such as controlled cross-linking or engineered composite systems with tailored degradation rates.

## 5. Conclusions

The study confirms the biocompatibility and osteogenic potential of HA/TTCP-based ceramic granules in a sheep bone defect model. Alginate coating improves handling and extrudability, though it may impair biological performance. The presence of oBMC was associated with a more favorable bone response in uncoated formulations, suggesting that the interplay between the biological and the physicochemical/structural characteristics of the ceramic formulation may modulate the osteogenic response. This study supports the continued development of injectable ceramic composites as adaptable, bioactive solutions for bone regeneration, emphasizing the importance of tailoring degradation profiles and material interfaces to optimize clinical outcomes.

## Data Availability

The original contributions presented in the study are included in the article/Supplementary Materials; further inquiries can be directed to the corresponding author.

## Supplementary Materials

The following supporting information can be downloaded at: https://www.mdpi.com/article/10.3390/jfb17090456/s1, Table S1: Experimental design and allocation of formulations to implantation sites.

## Abbreviations

The following abbreviations are used in this manuscript:

HA/TTCP	hydroxyapatite/tetracalcium phosphate
oBMC	ovine bone marrow concentrate
CaP	calcium phosphate
β-TCP	β-tricalcium phosphate
HA	hydroxyapatite
MNCs	mononuclear cells
α-MEM	α-modified minimum essential medium
FBS	fetal bovine serum
DT	doubling time
PD	population doublings
CPD	cumulative population doubling
CFU	colony-forming unit
ROI	regions of interest
BIC	bone-to-implant contact
BV/TV	trabecular bone volume
Mat.V/TV	material volume
MAR	mineral apposition rate
MS/BS	mineralizing surface
BFR/BS	bone formation rate
SD	standard deviation
MSC	mesenchymal progenitor cells
