# Supplementary material for: Injectable Hydroxyapatite and Tetracalcium Phosphate Granules Combined with Autologous Bone Marrow Concentrate: Toward Shape-Adaptive, Single-Step Bone Regeneration
Source: J Funct Biomater. 2026 Sep 8;17(9):456. doi: 10.3390/jfb17090456 (PMC13608013; doi:10.3390/jfb17090456)
Supplement: Supplementary file 1 [file jfb-17-00456-s001.zip › jfb-4460700-supplementary.pdf]

Table S1. Experimental design and allocation of formulations to implantation sites.

**Time point**	**Animal**	**Right femoral condyle**	**Left femoral condyle**	**Right tibial plateau**	**Left tibial plateau**
6 weeks	1	3 – Gel-coated HA/TTCP + oBMC	2 – Uncoated HA/TTCP	1 – Uncoated HA/TTCP + oBMC	4 – Gel-coated HA/TTCP
	2	4 – Gel-coated HA/TTCP	2 – Uncoated HA/TTCP	2 – Uncoated HA/TTCP	4 – Gel-coated HA/TTCP
	3	4 – Gel-coated HA/TTCP	4 – Gel-coated HA/TTCP	1 – Uncoated HA/TTCP + oBMC	1 – Uncoated HA/TTCP+ oBMC
	4	1 – Uncoated HA/TTCP + oBMC	2 – Uncoated HA/TTCP	3 – Gel-coated HA/TTCP + oBMC	3 – Gel-coated HA/TTCP + oBMC
12 weeks	5	3 – Gel-coated HA/TTCP + oBMC	4 – Gel-coated HA/TTCP	1 – Uncoated HA/TTCP + oBMC	2 – Uncoated HA/TTCP
	6	2 – Uncoated HA/TTCP	3 – Gel-coated HA/TTCP + oBMC	4 – Gel-coated HA/TTCP	1 – Uncoated HA/TTCP + oBMC
	7	1– Uncoated HA/TTCP + oBMC	2 – UnGel-coated HA/TTCP	3 –Gel-coated HA/TTCP + oBMC	4 – Gel-coated HA/TTCP
	8	2 – Uncoated HA/TTCP	3 – Gel-coated HA/TTCP + oBMC	4 – Gel-coated HA/TTCP	1 – Uncoated HA/TTCP + oBMC
